# Quantitative Study of Cell Invasion Process under Extracellular Stimulation of Cytokine in a Microfluidic Device

**DOI:** 10.1038/srep25557

**Published:** 2016-05-06

**Authors:** Kin Fong Lei, Hsueh-Peng Tseng, Chia-Yi Lee, Ngan-Ming Tsang

**Affiliations:** 1 Graduate Institute of Medical Mechatronics, Chang Gung University, Taoyuan, Taiwan; 2 Department of Mechanical Engineering, Chang Gung University, Taoyuan, Taiwan; 3 Department of Radiation Oncology, Chang Gung Memorial Hospital, Linkou, Taiwan; 4 Ph.D. Program in Biomedical Engineering, Chang Gung University, Taoyuan, Taiwan; 5 School of Traditional Chinese Medicine, Chang Gung University, Taoyuan, Taiwan

## Abstract

Cell invasion is the first step of cancer metastasis that is the primary cause of death for cancer patients and defined as cell movement through extracellular matrix (ECM). Investigation of the correlation between cell invasive and extracellular stimulation is critical for the inhabitation of metastatic dissemination. Conventional cell invasion assay is based on Boyden chamber assay, which has a number of limitations. In this work, a microfluidic device incorporating with impedance measurement technique was developed for quantitative investigation of cell invasion process. The device consisted of 2 reservoirs connecting with a microchannel filled with hydrogel. Malignant cells invaded along the microchannel and impedance measurement was concurrently conducted by measuring across electrodes located at the bottom of the microchannel. Therefore, cell invasion process could be monitored in real-time and non-invasive manner. Also, cell invasion rate was then calculated to study the correlation between cell invasion and extracellular stimulation, i.e., IL-6 cytokine. Results showed that cell invasion rate was directly proportional to the IL-6 concentration. The microfluidic device provides a reliable and convenient platform for cell-based assays to facilitate more quantitative assessments in cancer research.

Cancer metastasis is a cancer that has spread from one part of the body (primary site) to another not directly connected with it. It represents the major problem in the cancer treatment and has dramatic effects on the survival of patients. To achieve metastasis, malignant tumor cells should penetrate tissue barriers, such as the basement membrane, migrate through blood or lymph vessels, and rise of distant colonies^1^. Cell invasion is the first step of metastasis and defined as cell movement through extracellular matrix (ECM) which requires adhesion, proteolysis of ECM, and migration^2^. Therefore, investigation of the basic principles and molecular pathways of cell invasion is critical to inhibit metastatic dissemination. For example, increase of interleukin-6 (IL-6) cytokine leads to increase the rate of malignancy and promote cancer metastasis^3^,^4^,^5^,^6^. When IL-6 engages the receptor of cells, a number of cellular phosphorylation and signaling pathways are triggered. It results a wide range of cellular processes including cell proliferation, oncogenesis, and cancer metastasis^7^. Understanding of the correlation between cell invasion and extracellular stimulation, e.g., cytokine, is essential to study cell metastasis and thus develop effective therapeutic strategies for controlling invasive malignant tumor cells.

Currently, most of cell invasion assays are based on Boyden chamber assay in biological laboratory. A transwell coating with a layer of ECM on membrane is used and cells move through the ECM to study the cell invasion process. Hence, invaded cells can be stained and quantified on the membrane under microscope. The Boyden chamber assay is widely used but also has inherent limitations. The pore size of the membrane highly influences the number of invaded cells. Also, since cells move from the upper transwell to the lower culture chamber, cell invasion may be induced by gravity. Moreover, this assay is an end-point assay and the quantification of invaded cells is subjective. These are the major concerns of the Boyden chamber assay and development of alternative methods for cell invasion assay becomes necessary.

In the past decades, development of microfluidic technology becomes mature and a lot of biomedical applications have been demonstrated on microfluidic systems^8^,^9^,^10^. For cell-based assays in microfluidic systems, one of the important advantages is to provide a well-controlled environment for precise study of cellular activities^11^,^12^,^13^,^14^. By designing special microchannels, cell invasion could be observed in the microfluidic systems^15^,^16^,^17^,^18^. For example, a microfluidic device was developed for monitoring cell migration across ECM-coated microgaps^15^. Migration of invasive MDA-MB-231 cells was tracked by real-time light microscopy. Alternatively, transendothelial invasion of tumor aggregates was successfully observed in a microfluidic system^18^. Adenoid cystic carcinoma cell aggregates transmigrated across the endothelium under the stimulation of chemokine CXCL12 and the invasion was inhibited by CXCR4 antagonist. This *in vitro* device allowed for detailed study of the attachment and transendothelial invasion of tumor aggregates. In the above excellent demonstrations, observation of cell invasion activity was based on imaging under optical microscope. Results were achieved by capturing and comparing the images at the beginning and at regular intervals during cell invasion process. However, quantitative correlation between cell invasion and extracellular stimulation is challenging to be evaluated by optical imaging. In order to achieve quantitative study of cell invasion under extracellular stimulation, in this work, a microfluidic device incorporating with impedance measurement technique was developed to provide real-time, non-invasive, and objective investigation of cellular activities.

A microfluidic device with 2 reservoirs connecting with a microchannel was developed. Malignant cells (cell line: NPC-BM1) were seeded in one reservoir and invaded to another reservoir through the microchannel filled with methyl cellulose (MC) hydrogel. The MC hydrogel was used to mimic the basement membrane *in vivo*. On the bottom surface of the microchannel, eight electrodes were embedded for impedance measurement. Cells moving along the microchannel could be monitored in a real-time and non-invasive manner. Hence, cell invasion rate could be calculated by the impedance change of the electrodes. Influence of cell invasion rate under stimulation of cytokine, i.e., IL-6, was demonstrated to quantitatively study the correlation between cell invasion and extracellular stimulation. As a whole, the current microfluidic device provides a platform to study cell invasion process without influences of pore size of the membrane and gravity that are the major concerns in Boyden chamber assay. Moreover, quantitative monitoring of cell invasion process was successfully achieved by impedance measurement technique. This microfluidic device is proposed to be a convenient and quantitative tool for cell invasion assay.

## Results and Discussion

### Design of the microfluidic device for monitoring of cell invasion process

**Figure 1:**
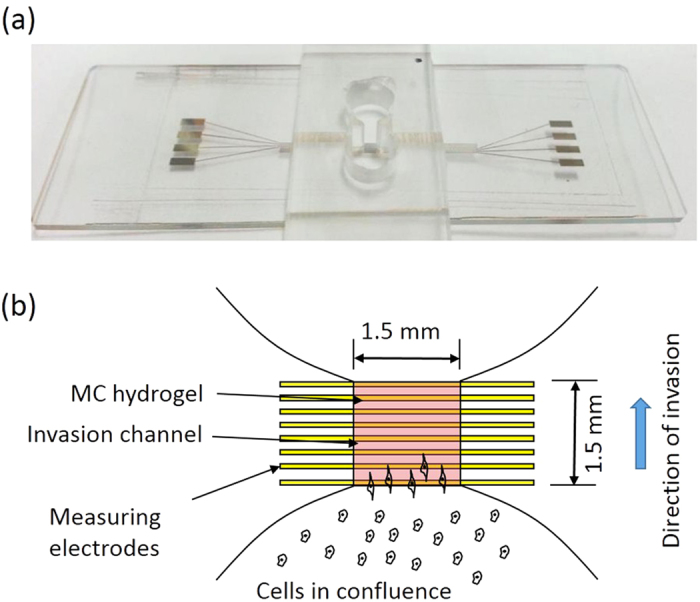
Microfluidic device for impedimetric monitoring of cell invasion process. (**a**) Photograph of the microfluidic device. (**b**) Schematic illustration of the principle of monitoring the cell invasion process.

**Figure 2:**
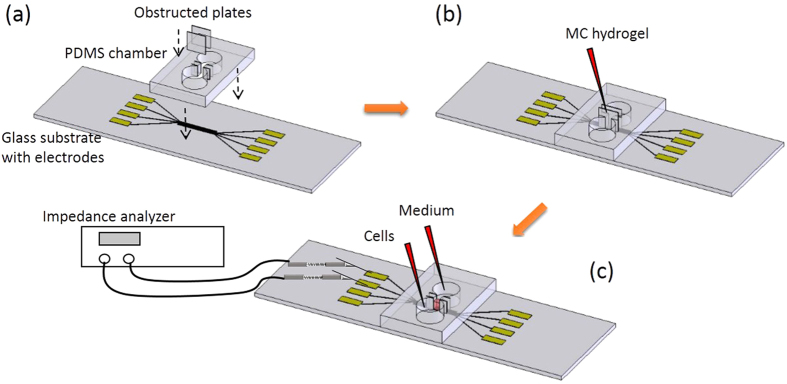
Experimental procedures of the cell invasion assay in the microfluidic device. (**a**) Assembly of the microfluidic device. (**b**) Loading and solidification of MC hydrogel in the microchannel. (**c**) Loading of cells in serum-free medium in one reservoir and medium with 20% FBS in another reservoir. The microfluidic device was then transferred in an incubator. Impedance measurement was conducted by measuring each pair of electrodes using an impedance analyzer.

A microfluidic device incorporating with impedance measurement technique was developed to quantitatively study of cell invasion process under the stimulation of cytokine. The device was composed of a glass substrate embedded with 8 straight electrodes and a polydimethylsiloxane (PDMS) layer with 2 reservoirs connecting with a microchannel. Photograph of the microfluidic device and the principle of monitoring the cell invasion process are shown in Fig. 1. The electrodes were aligned vertically with the microchannel for the impedance measurement. The experimental procedures of the cell invasion assay in the microfluidic device is shown in Fig. 2. Before cell invasion experiment, the microchannel was filled with MC hydrogel to mimic the basement membrane *in vivo*. Cells were seeded in one reservoir and invaded to another reservoir through the microchannel. During the process, cells moved along the microchannel and the total impedance across each pair of electrodes increased to determine the presence of cells. Because the distance of electrodes was known, the impedance change of each pair of electrodes represented the location and number of cells. Hence, cell invasion rate could be calculated by continuous impedance measurement of each pair of electrodes at different fixed distances.

### Optimization of the impedance measurement conditions

**Figure 3:**
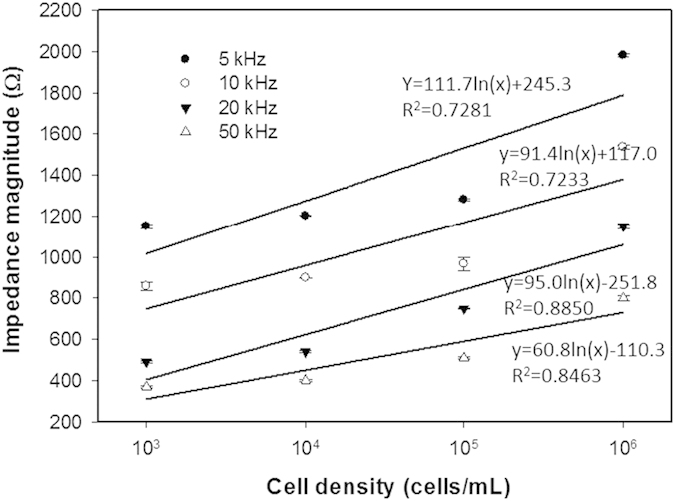
Correlation between impedance magnitude and cell density under different measuring frequencies of 5, 10, 20, and 50 kHz. Error bars represent the standard errors of 3 repeated experiments. The solid lines represent the results of linear regressive approximation.

Because cell membrane is modeled as an equivalent electrical circuit of capacitance and resistance in series, the impedance magnitude of cells attaching on the electrode surface is varied by the measuring frequency^19^. When the frequency is sufficient low or high, sensitivity of the measurement of cellular behaviors becomes extremely low. Also, the frequency may be varied by different culture environments and measuring electrodes. Therefore, optimization of the measuring frequency was investigated in order to figure out the highest sensitivity and linearity between impedance magnitude and cell density in the microfluidic device. Cells at different densities of 10^3^, 10^4^, 10^5^, 10^6^, and 10^7^ cells/mL were respectively introduced to the microchannel blocked by 2 obstructed plates at its ends. After 8 hr for cell attachment, MC hydrogel was applied to the microchannel. Hence, impedance magnitude across the electrodes located at the bottom of the microchannel was measured and analyzed. Correlation between impedance magnitude and cell density under different measuring frequencies of 5, 10, 20, and 50 kHz is shown in Fig. 3. Generally, cell density was found to be proportional to the impedance magnitude at all measuring frequencies. The sensitivity values, i.e., slope of the correlation, were respectively 111.7, 91.4, 95.0, and 60.8, and the linearity values, i.e., R-squared values, were respectively 0.7281, 0.7233, 0.8850, and 0.8463. Measurement at 20 kHz was found to have the highest linear correlation with reasonable sensitivity. Hence, the measuring frequency of 20 kHz was identified and used in the following experiments in this study.

### Real-time and non-invasive impedimetric monitoring of cell invasion process

**Figure 4:**
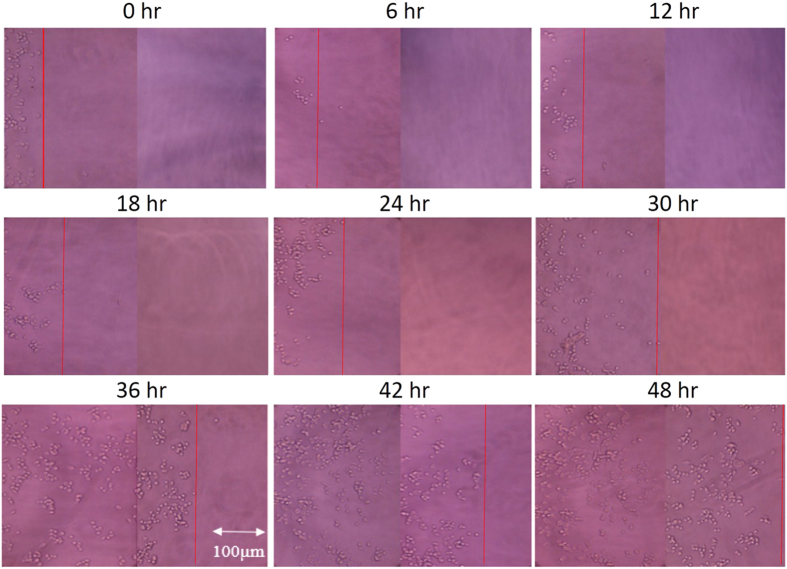
Successive microscopic images of cell invasion process. The red lines represents the leading edges of cells at the corresponding time points.

**Figure 5:**
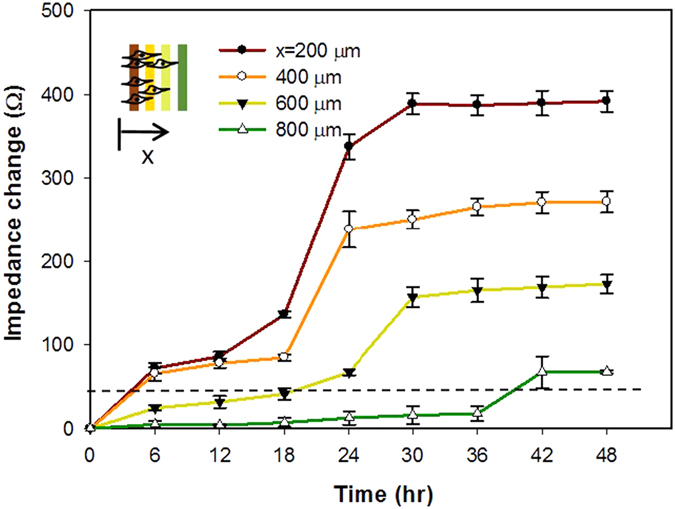
Impedance measurements across the electrodes at the distances of 200, 400, 600, and 800 μm at different successive time points. Error bars represent the standard errors of 3 repeated experiments. The dash line represents the impedance change of 32 Ω, i.e., a threshold of certain cells reaching the electrode surface.

In order to confirm the applicability of the microfluidic device for cell invasion assay, experiment of cell invasion was performed and results were observed under microscope. Noted that the microchannel was not embedded with the electrodes in order to have clear microscopic images. During the process, cells invaded from one reservoir to another reservoir through the connecting microchannel filled with MC hydrogel. Microscopic images of the microchannel were captured every 6 hr and is shown in Fig. 4. The leading edges of cells at different successive time points were manually identified from the images. Based on linear regressive approximation, cell invasion rate was estimated to be 12.6 μ m/h. Results showed that the microfluidic device is feasible to perform cell invasion assay with the advantages of without influences of pore size of the membrane and gravity that are the major concerns in Boyden chamber assay. The quantification based on microscopic observation was direct and convenient; but it was subjective. Therefore, to provide an objective approach, cell invasion process was then monitored by impedance measurement across 8 straight electrodes embedded on the bottom of the microchannel. Results of impedance measurement at different successive time points are shown in Fig. 5. Microscopic images correlating to the experimental data shown in Fig. 5 were included in Fig. S1 in the supplementary materials. Because cells reached the distance of 800 μ m in the experiment within 48 hr, only impedance changes of the electrodes located at the distances of 200, 400, 600, and 800 μ m were shown in the figure. Increase of impedance change represented cell attachment on the electrode surface. Results showed that cells were progressively invaded from the reservoir until the distance of 800 μ m. Cell invasion process was quantitatively monitored in the microfluidic device. In order to calculate the cell invasion rate, we defined the impedance change of 32 Ω as a threshold of certain cells reaching the electrode surface. Because environmental noise may influence the impedance measurement, we found that impedance change over 32 Ω significantly indicated cell attachment on the electrode surface (data not shown). Based on linear regressive approximation, cell invasion rate was calculated to be 10.0 μ m/h.

In this work, results shown in Fig. 4 (microscopic observation) and Fig. 5 (impedance measurement) were 2 independent experiments. In cell biological study, experimental data are only compared in the same experiment under same condition. The most differences of different experiments are cell conditions, e.g., generation, cell cycle, and source, which cannot be controlled. Therefore, cell invasion rates calculated from 2 approaches could not be directly compared but they still reasonably matched.

### Cell invasion under the stimulation of cytokine

**Figure 6:**
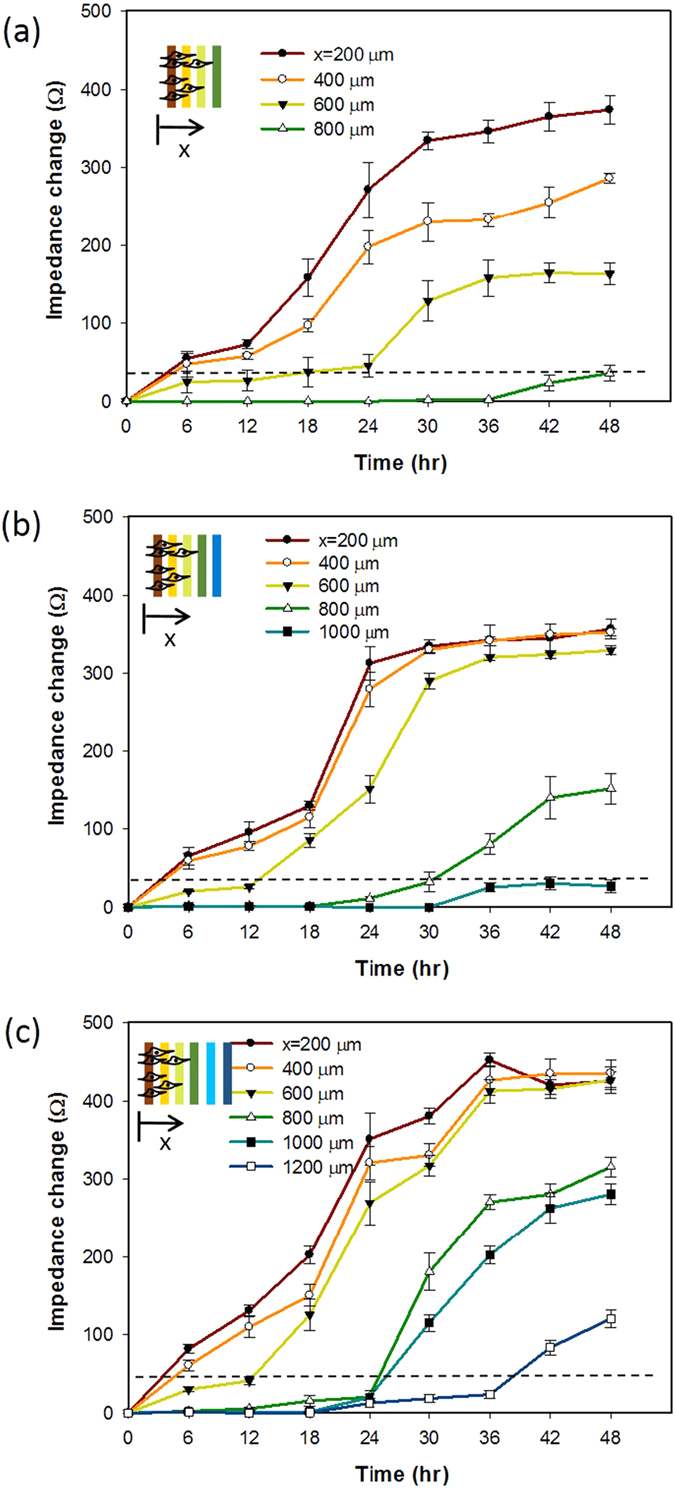
Impedimetric monitoring of cell invasion process under the stimulation of IL-6 cytokine at the concentrations of (**a**) 2, (**b**) 5, and (**c**) 10 ng/mL. Error bars represent the standard errors of 3 repeated experiments. The dash line represents the impedance change of 32 Ω, i.e., a threshold of certain cells reaching the electrode surface.

Understanding of the correlation between cell invasion rate and extracellular stimulation, e.g., cytokine, is essential to study cell metastasis for the development of effective therapeutic strategies. Cytokine is one of the interesting topics of influencing cancer pathogenesis^20^,^21^,^22^. It was reported that cells infected by Epstein-Barr virus lead to the oncogenic transformation^23^,^24^ and induce the production of cytokines such as IL-6 and IL-10^25^,^26^. The production of IL-6 from virus-transformed cells affects the neighboring cells to increase the rate of malignancy and promote cancer metastasis^3^,^4^,^5^,^6^. Such that, cell invasion rate could be promoted by the stimulation of cytokine. To quantitatively study this correlation, cells were respectively stimulated by IL-6 at different concentrations and the corresponding cell invasion rate was then calculated. Results of the impedance measurements are shown in Fig. 6. Analysis of percentage of invaded cells was included in Fig. S2 in the supplementary materials. Cell invasion rates under IL-6 concentrations of 2, 5, and 10 ng/mL were calculated to be 10.3, 12.2, and 20.3 μ m/h, respectively. Generally, cell invasion rate was directly proportional to the IL-6 concentration. It was reported that higher activation level of transcription factors is triggered by higher concentration of IL-6^27^. Results confirmed that the microfluidic device could quantitatively monitor the entire cell invasion process.

## Conclusion

A microfluidic device incorporating with impedance measurement technique was developed for the investigation of cell invasion process. Cells invaded along the microchannel filled with hydrogel and the process could be monitored in real-time and non-invasive manner. Moreover, quantitative study of the correlation between cell invasion rate and extracellular stimulation was achieved. Results showed that cell invasion rate was directly proportional to the IL-6 concentration. In conclusion, reliable assessment of cellular activity is very important for the development of effective therapeutic strategies. Design of the microfluidic device was to eliminate the influences of membrane and gravity that are the major concerns in Boyden chamber assay. Also, the microfluidic device provides a promising tool to quantitatively study cell invasion process under extracellular stimulation. This technology has potential to be developed to a reliable and convenient analytical platform for facilitating quantitative assessments in cancer research.

## Materials and Methods

### Cell culture

An epithelial cell line (NPC-BM1) derived from a bone marrow biopsy of a female Taiwanese patient with nasopharyngeal carcinoma (NPC) was kindly provided by Dr. Jenny Liu, Chang Gung University. Culture medium was Dulbecco’s modified eagle medium (DMEM; Invitrogen, USA) supplemented with 10% fetal bovine serum (FBS; Invitrogen, USA) and antibiotic/antimycotic (100 U/mL of penicillin G sodium, 100 mg/mL of streptomycin, and 0.25 mg/mL of amphotericin B; Gibco-BRL Life Technologies, USA). Cells were amplified by standard cell culture technique and trypsinized using 0.05% trypsin (Gibco-BRL Life Technologies, USA) for 3 min, centrifuged at 1200 rpm for 5 min, and re-suspended in the medium for further experiments.

### Fabrication of the microfluidic device

The microfluidic device consisted of a glass substrate and a PDMS (Model: Sylgard^®^ 184; Dow Corning, USA) layer. Eight straight Cr/Au (50/500 Å) electrodes were fabricated on the glass substrate by standard microfabrication processes including thermal evaporation and lift-off. The electrodes were 100 μ m in width and the gap between electrodes was 100 μ m. The PDMS layer was fabricated by soft lithography and the fabrication process is briefly described. A poly(methyl methacrylate) (PMMA) mold with negative pattern of reservoirs and microchannel was machined by CNC engraving machine (Model: EGX-400; Roland, USA). Then, PDMS pre-polymer and curing agent in (w/w) 10:1 were mixed and poured to the PMMA mold. The PDMS was solidified at 70 °C for 1hr and then peeled off from the mold. Finally, the microfluidic device was completed by bonding the glass substrate and the PDMS layer using oxygen plasma (Model: PDC-32 G; Harrick Plasma, USA). Before conducting experiment, the microfluidic device was sterilized using 70% (w/v) ethanol for 6 h, washed by phosphate-buffered saline (PBS; 50 mM phosphate, 150 mM NaCl, and 10 mM EDTA; pH 7.6), and kept under ultraviolet light overnight.

### Cell invasion assay in the microfluidic device

The microfluidic device provides a platform for the investigation of cell invasion without the use of membrane and the influence of gravity. In order to form a barrier to mimic basement membrane *in vivo*, MC hydrogel was used in this study. It was prepared by dispersing MC powder (Methocel® MC; Sigma, USA) in PBS at a concentration of 3% (w/v) at room temperature and was sterilized by using autoclave at 121 °C under 100 kPa for 20 min for further use. The experimental procedures of the cell invasion assay in the microfluidic device is shown in Fig. 2. Two obstructed polycarbonate plates were inserted at 2 ends of the microchannel. Then, the MC hydrogel was loaded in the microchannel and a gel-like barrier was formed after solidification. Next, cells at cell density of 3 ×  10^5^ cells/mL in serum-free medium were applied to one reservoir. Culture medium with 20% FBS was applied to another reservoir for inducing cell invasion. The microfluidic device was then transferred in a 37 °C and 5% CO_2_ humidified incubator (Model: 370; Thermoscientific, USA). After 6 hr for cell seeding and spreading, the obstructed plates could be removed and the experiment was defined to be started (0 hr). Investigation of cell invasion without extracellular stimulation was first carried out up to 2 days. Result was a control to compare to the results with the stimulation of cytokine, i.e., IL-6 (Invitrogen, USA) in different concentrations. During these cell invasion experiments, impedance measurement across each pair of electrodes was concurrently conducted by using an impedance analyzer (Model: VersaSTAT 4; Princeton Applied Research, USA). Hence, real-time and non-invasive monitoring of cell invasion activity was demonstrated in the microfluidic device.

### Impedance measurement of cell invasion in the microfluidic device

A pioneer work of using impedance measurement technique for the cellular monitoring was reported in 1984^28^. Later, it has been widely demonstrated on the quantification of various cellular activities, i.e., proliferation, viability, physiological state of cells^29^,^30^,^31^,^32^,^33^,^34^. These demonstrations were based on using a pair of coplanar electrodes on a solid surface for the electrical transducer. Because cell membrane is lipid bilayer structure and has capacitance of 0.5–1.3 μ F/cm^2^ and resistance of 10^2^–10^5^ Ω/cm^2^ 35, cells spreading on the electrode surface can induce the impedance change across the electrodes. When cells attach onto the electrode surface, the total impedance across the electrodes can be modeled as capacitances and resistances in series, which reflects the biophysical properties of the insulating cell membrane. When the frequency is sufficient low or high, sensitivity of the cell impedance measurement becomes extremely low. It was suggested that the most appropriate frequency for cell impedance measurement is 20 kHz at which the sensor will be at the highest sensitivity^19^. Although the optimized frequency may vary from different device parameters, cell impedance measurement at medium frequencies (5–100 kHz) is a universal criteria. Moreover, it was reported that there was no detectable electrical influence on cells and the measurement can be used for long-term continuous monitoring^36^. Hence, the impedance measurement is generally believed that it can provide a convenient and reliable measurement for monitoring of cellular activities.

In this study, during the 2 day cell invasion experiment, impedance measurement was performed every 6 hr. Potential of 0.1 Vrms was applied across the electrodes and the impedance was measured from 1 to 100 kHz. Eight electrodes were respectively located at the distances of 200, 400, 600, 800, 1000, 1200, 1400, and 1600 μ m. Seven measurements were respectively conducted by 7 pairs of electrodes located at 200 and 400 μ m, 400 and 600 μ m, 600 and 800 μ m, 600 and 1000 μ m, 1000 and 1200 μ m, 1200 and 1400 μ m, and 1400 and 1600 μ m. Therefore, seven impedance values were collected for each measurement at successive time point. In order to eliminate the variations between electrodes, background impedance value of each pair of electrodes was measured before loading of cells. Impedance change was defined as the subtraction of the impedance value at the successive time point and the background impedance value.

## Additional Information

**How to cite this article**: Lei, K. F. *et al.* Quantitative Study of Cell Invasion Process under Extracellular Stimulation of Cytokine in a Microfluidic Device. *Sci. Rep.* **6**, 25557; doi: 10.1038/srep25557 (2016).

## Supplementary Material

Supplementary Information
