# Supplementary material for: Quantitative Study of Cell Invasion Process under Extracellular Stimulation of Cytokine in a Microfluidic Device
Source: Sci Rep. 2016 May 6;6:25557. doi: 10.1038/srep25557 (PMC4858876; doi:10.1038/srep25557)
Supplement: Supplementary Information [file srep25557-s1.pdf]

Supplementary Materials

Quantitative Study of Cell Invasion Process under Extracellular Stimulation of Cytokine in a Microfluidic Device

Kin Fong Lei [1, 2, 3, *], Hsueh-Peng Tseng [1], Chia-Yi Lee [4], Ngan-Ming Tsang [3, 5]

[1] Graduate Institute of Medical Mechatronics, Chang Gung University, Taoyuan, Taiwan

[2] Department of Mechanical Engineering, Chang Gung University, Taoyuan, Taiwan

[3] Department of Radiation Oncology, Chang Gung Memorial Hospital, Linkou, Taiwan

[4] Ph.D. Program in Biomedical Engineering, Chang Gung University, Taoyuan, Taiwan

[5] School of Traditional Chinese Medicine, Chang Gung University, Taoyuan, Taiwan

[*] E-mail: kflei@mail.cgu.edu.tw

Microscopic images correlating to the experimental data shown in Fig. 5

In order to provide more information, microscopic images correlating to the experimental data shown in Fig. 5 are shown in Fig. S1. The leading edges of cells at different successive time points were identified from the images. Based on linear regressive approximation, cell invasion rate was estimated to be 10.5 μm/h, which has no significant difference with the invasion rate calculated by impedance measurement from Fig. 5.

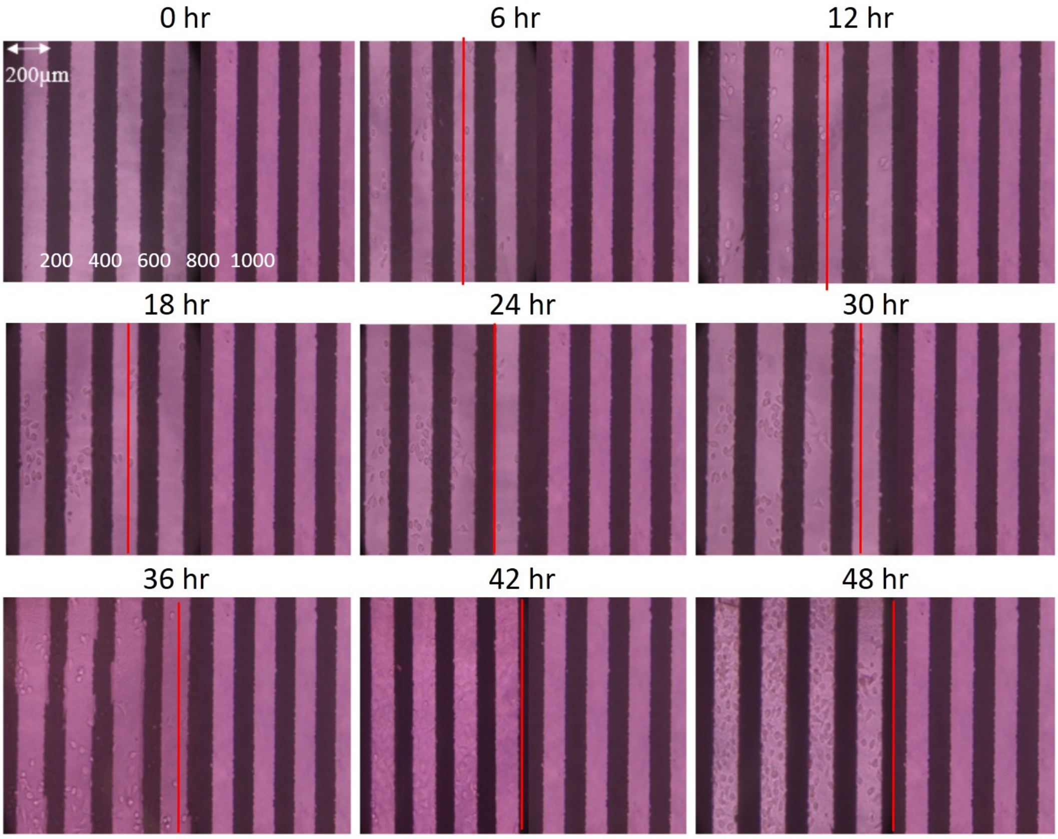

Fig. S1. Successive microscopic images correlating to the experimental data shown in Fig. 5. The red lines represents the leading edges of cells at the corresponding time points.

Analysis of percentage of invaded cells under the stimulation of IL-6 cytokine

Analysis of percentage of invaded cells under the stimulation of IL-6 cytokine at the concentration of 0, 2, 5, and 10 ng/mL is shown in Fig. S2. Results indicated cells invaded to longer distance when higher concentration of IL-6 cytokine was applied.

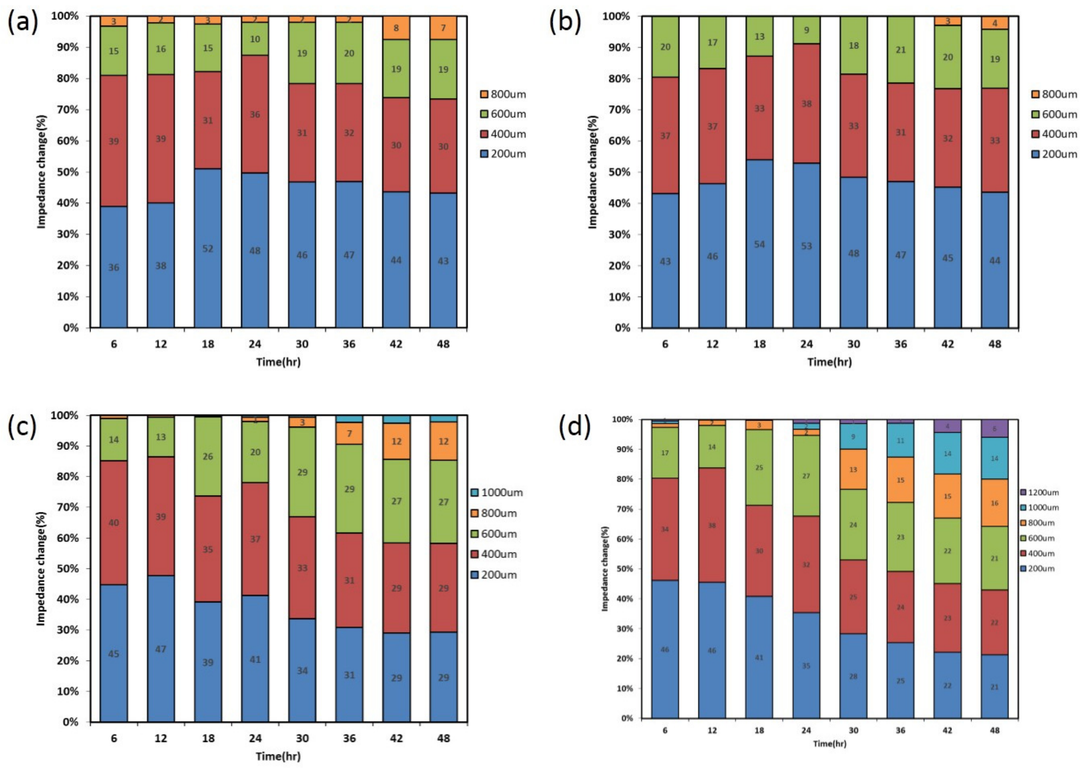

Fig. S2. Analysis of percentage of invaded cells under the stimulation of IL-6 cytokine at the concentrations of (a) 0, (b) 2, (c) 5, and (d) 10 ng/mL.
